# Exact and fast calculation of the X-ray pair distribution function

**DOI:** 10.1107/S1600576720004616

**Published:** 2020-05-13

**Authors:** Reinhard B. Neder, Thomas Proffen

**Affiliations:** a Kristallographie und Strukturphysik, Friedrich-Alexander-Universität Erlangen–Nürnberg, Staudtstrasse 3, 91058 Erlangen, Germany; b Neutron Scattering Division, Oak Ridge National Laboratory, Oak Ridge, TN 37831, USA

**Keywords:** pair distribution function, PDF, powder diffraction

## Abstract

A fast and exact algorithm to calculate the powder pair distribution function (PDF) for the case of periodic structures is presented. The new algorithm calculates the PDF by a detour via reciprocal space. The calculated normalized total powder diffraction pattern is transferred into the PDF via the sine Fourier transform. The calculation of the PDF via the powder pattern avoids the conventional simplification of X-ray and electron atomic form factors. It is thus exact for these types of radiation, as is the conventional calculation for the case of neutron diffraction. The new algorithm further improves the calculation speed. Additional advantages are the improved detection of errors in the primary data, the handling of preferred orientation, the ease of treatment of magnetic scattering and a large improvement to accommodate more complex instrumental resolution functions.

## Abstract 2

A fast and exact algorithm to calculate the powder pair distribution function (PDF) for the case of periodic structures is presented. The algorithm especially improves X-ray and electron PDF calculations, and the handling of instrumental resolution functions.

## 1. Introduction   

The powder pair distribution function (PDF) is commonly used to characterize the local structure of a wide range of materials like disordered crystalline matter, nanoparticles, and amorphous materials including glasses and liquids (Egami & Billinge, 2012 ▸; Young & Goodwin, 2011 ▸; Playford *et al.*, 2014 ▸; Mancini & Malavasi, 2015 ▸). Originally developed for the analysis of disordered bulk materials, the method is nowadays very widely used for the analysis of nanoparticles as well, with numerous publications. For an early application see Korsunskiy & Neder (2005 ▸) and Neder & Korsunsky (2005 ▸). While predominantly used with neutron and X-ray diffraction experiments, more recently it has also been used with electron diffraction (Abeykoon *et al.*, 2019 ▸, 2015 ▸; Gorelik *et al.*, 2019 ▸). Other technical developments include the application to magnetic short-range order (Frandsen *et al.*, 2014 ▸) and thin films (Jensen *et al.*, 2015 ▸; Shi *et al.*, 2017 ▸; Dippel *et al.*, 2019 ▸). The main application is still in the field of static structural characterization; for the field of dynamic structure characterization see Egami & Billinge (2012 ▸).

The PDF is obtained from a powder diffraction experiment after suitable normalization, division by the average atomic form factor, and correction for background and further experimental aspects. This correction converts the powder diffraction intensity to the normalized total scattering function *S*(*Q*), with *Q* = 4πsin(θ)/λ, where θ is half the scattering angle and λ is the wavelength of the incident radiation. The widely used reduced PDF *G*(*r*) is obtained from *S*(*Q*) via a sine Fourier transformation:
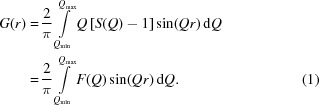
Various other definitions of the PDF exist [see Keen (2001 ▸) for a review]. In this article we will always refer to the reduced PDF *G*(*r*).

The sine Fourier transform that is used in equation (1) converts the normalized intensity into direct space. The PDF is thus an extension of the Patterson function, which is also a Fourier transform of the observed intensities. As the classical Patterson function uses integrated Bragg intensities only, its calculation reduces to a Fourier series rather than the Fourier integral of equation (1) and the Patterson function is inherently periodic in 3D space. Since the Fourier integral in equation (1) includes all contributions in reciprocal space, Bragg data and the diffuse scattering, the PDF is no longer a periodic function in direct space. Still, the PDF as obtained via equation (1) is essentially a histogram of the interatomic distances weighted by the scattering power of the pair of atoms. Thus, the use of different types of radiation like neutrons, X-rays or electrons yields a PDF in which the relative contributions of light and heavy elements or different isotopes will differ according to the strength of the interaction between the radiation and the atom types present in the sample.

Conventionally, the model PDF is determined from a structural model by summing all interatomic distances. This algorithm is used in common analysis programs such as *DISCUS* (Proffen & Neder, 1997 ▸; Neder & Proffen, 2008 ▸), *PDFgui* (Farrow *et al.*, 2007 ▸), *RMCprofile* (Tucker *et al.*, 2007 ▸) and *TOPAS* (Bruker, 2015 ▸; Coelho, 2018 ▸). As detailed in the next section, this algorithm is only exact in the case of neutron diffraction. Since this algorithm approximates the influence of the *Q*-dependent atomic form factor in the case of X-ray and electron diffraction by a constant number, it represents a simplification that has been known for a long time (Warren *et al.*, 1936 ▸). To our knowledge, all currently available software uses this Warren–Krutter–Morningstar approximation. A common refinement of data in direct and reciprocal space will lessen the approximation, a notable recommendation mentioned in the *RMCprofile* manual. Few attempts have been made to take this *Q* dependence into consideration (Korsunskiy & Neder, 2005 ▸; Masson & Thomas, 2013 ▸). In the latter paper an exact expression for the calculation of the PDF in direct space is derived by splitting the PDF into a linear combination of modified partial PDFs.

In this article, a new algorithm to calculate the PDF is introduced. This algorithm no longer relies on the approximation of the atomic form factor. Instead, the PDF is calculated by a detour via a calculation of the powder pattern. As this calculation correctly takes the *Q* dependence of the atomic form factors into account, it is thus an exact calculation of the powder PDF for neutron, X-ray and electron diffraction. Within the article we focus on the calculation of the PDF for a bulk sample. This mostly corresponds to the capabilities included in the *PDFgui* software. The calculation of the PDF via the powder pattern requires a detailed calculation of the powder pattern in the case of disordered structures. Two different approaches are outlined here. The application of the algorithm and modifications of it with respect to arbitrarily shaped finite-sized nanoparticles will be presented in a forthcoming publication.

## 2. Traditional calculation of the PDF from a model structure   

For the case of a PDF obtained from a neutron diffraction experiment on bulk samples, the model PDF is readily calculated for a structure model by summing over all pairwise interatomic distances:
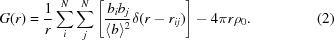
Here the sum runs over all atoms *i*, *j* in the model structure, separated by a distance *r*_*ij*_. The terms *b*_*i*_ and *b*_*j*_ are the coherent neutron scattering lengths of the atoms *i* and *j*, respectively, and 〈*b*〉^2^ is the squared average coherent neutron scattering length. Finally, ρ_0_ is the average number density in atoms per unit volume. The thermal motion of atoms can be modelled by convolving the histogram with a distribution function taken as a Gaussian distribution. Its width is determined by the combined atomic displacement parameters of the two atom types involved.

As it stands, equation (2) is correct for the case of neutron diffraction and a perfect instrument, whose resolution function is a delta distribution across all scattering angles and data collected up to *Q*_max_ = ∞. The effect of a finite *Q*_max_ value is incorporated into the PDF calculation by convolving the ideal PDF with the Fourier transform of the box function:
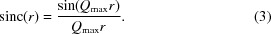



The effect of a finite resolution function is commonly approximated in the available computer programs by two instrument-dependent parameters, a damping term and a broadening term. The damping term is a multiplicative term 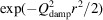, while the broadening term adds a distance-dependent component to the width of interatomic distance distributions. The calculations in *DISCUS* use the equation
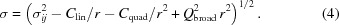
Here σ_*ij*_ is the width of the distribution that results from the independent thermal motion of atoms *i* and *j*, respectively. The terms *C*_lin_/*r* and *C*_quad_/*r* ^2^ allow for correction of the width of peaks in the PDF at short interatomic distances *r*. These terms approximate the correlated thermal motion of atoms at close distance, as present in an acoustic phonon. The last resolution-related term 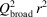 results in an approximately linear increase in the width of the interatomic distance distributions. The program *PDFgui* uses a slightly different equation,
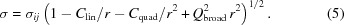



The two instrumental terms *Q*_damp_ and *Q*_broad_ are obtained by refining the model function with respect to the experimental PDF of a highly crystalline material like CeO_2_, Si, Ni or LaB_6_. In the *PDFgui* notation the displacement parameters of the atoms multiply all terms in the root, resulting in different numerical values for *Q*_broad_ compared with *DISCUS*. Further differences in numerical values will result if data for a series of different temperatures are compared. The instrumental parameter *Q*_broad_ has usually been determined by a refinement with respect to a standard sample at room temperature. If this fixed value is subsequently used in equations (4) or (5) for data measured at different temperatures, the numerical values for the sample atomic displacement parameters and their temperature dependence will differ.

## 3. Shortcomings of the traditional PDF calculations   

As mentioned in the previous section, the PDF calculation according to equation (2) is only exact for the case of neutron diffraction. For X-ray or electron scattering the scattering lengths are replaced by the atomic form factors evaluated at a fixed *Q* = *Q*_f_:
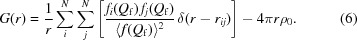
In many calculations the fixed value of *Q* is taken as *Q*_f_ = 0 Å^−1^, resulting in the ordinal number for a neutral atom. Alternatively, a value of *Q*_f_ = *Q*_max_/2 is recommended. The justification behind this simplification to use a fixed *Q* value is the assumption that the value of the quotient *f*_*i*_(*Q*) *f*_*j*_(*Q*)/〈*f*(*Q*)〉^2^ is reasonably constant across the entire *Q* range. This approximation is known as the Warren–Krutter–Morningstar approximation (Warren *et al.*, 1936 ▸). In their original paper Warren and co-workers used an effective number of electrons per atom as the approximation to the quotient of the individual atomic form factor divided by an average atomic form factor.

**Figure 1:**
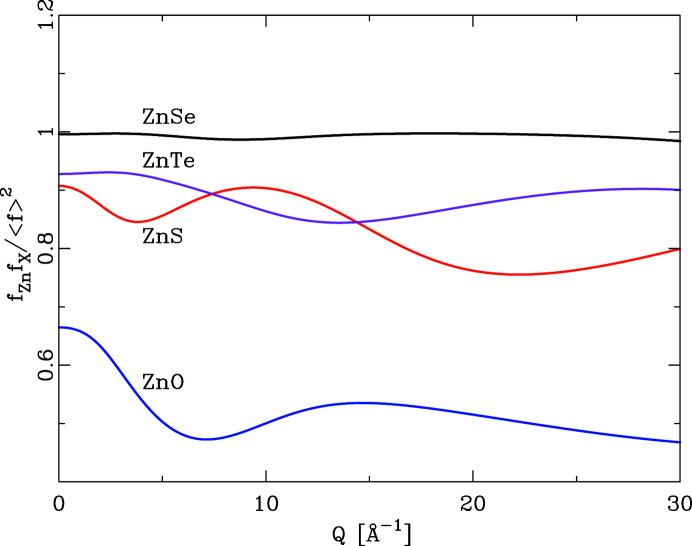
The *Q* dependence of *f*_*i*_ *f*_*j*_/〈*f*〉^2^ for different Zn*X* compounds. With the exception of ZnSe, these curves are not negligibly flat.

**Figure 2:**
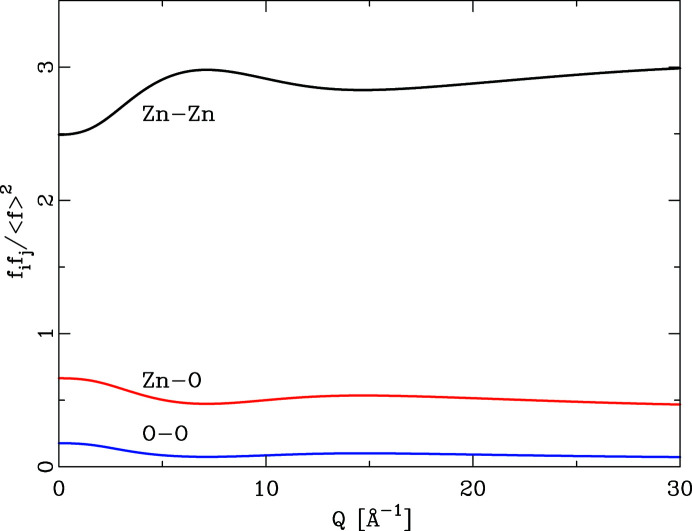
The *Q* dependence of *f*_*i*_ *f*_*j*_/〈*f*〉^2^ for the partial contributions Zn—Zn, Zn—O and O—O for ZnO. The *Q* dependence differs for the three partial contributions.

**Figure 3:**
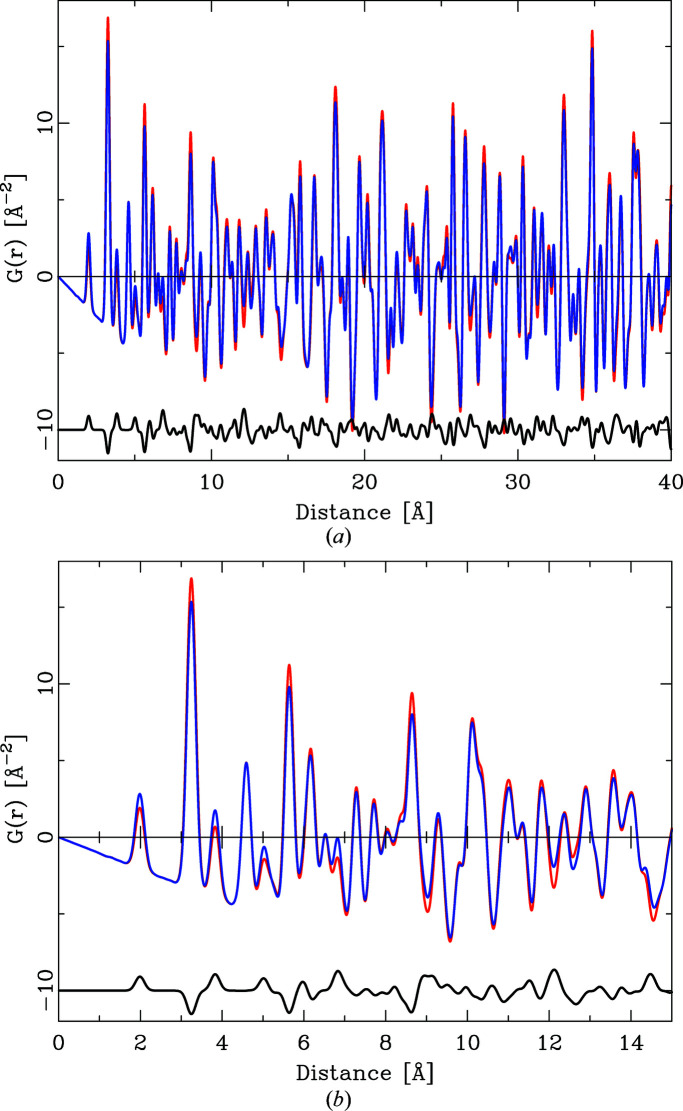
(*a*) The effect of *Q* choice for X-ray atomic form factors on the calculated PDF. The red PDF (background) was calculated for ZnO using *Q* = 7 Å^−1^ and the blue PDF for *Q* = 0 Å^−1^. The black difference curve reflects the difference PDF(*Q* = 0 Å^−1^) − PDF(*Q* = 7 Å^−1^), offset for clarity. (*b*) Detail of the graph in panel (*a*) in the distance range 0–15 Å.

Fig 1 ▸ shows the *Q* dependence of this quotient for the series ZnO, ZnS, ZnSe, ZnTe. With the exception of ZnSe, the *Q* dependence of all quotients is by no means constant. This has the effect that the integral heights of peaks in a PDF calculated by equation (6) will vary systematically if different *Q* values are chosen. As Fig. 2 ▸ further illustrates, the *Q* dependence of the different partial pair contributions to the PDF differs as well. Thus, if the PDF is calculated using different *Q* values for the atomic form factors in equation (6), the relative integral peak heights of these pair–pair correlations will change. This is demonstrated in Fig. 3 ▸ for a model of crystalline ZnO. The main graph shows PDFs calculated according to equation (6) using *Q* = 0 Å^−1^ for all atomic form factors (blue) and for *Q* = 7 Å^−1^ (red). The difference curve below shows the difference between the calculations using *Q* = 0 Å^−1^ and *Q* = 7 Å^−1^. Very significant differences between these two PDFs are obvious at all peak positions. The agreement between PDF(*Q* = 0 Å^−1^) and PDF(*Q* = 7 Å^−1^) is not very good, at an unweighted *R* value of 10%. As current model calculations aim to achieve a difference between model and calculated PDF that is much less than the difference shown in Fig. 3 ▸, this effect can no longer be neglected.

A second disadvantage of the PDF calculation according to either equation (2) or (6) is the dependence of the computational time on the distance range to be calculated. Due to the double sum over all atom pairs, the computational time is proportional to the square of *r*_max_.

A further disadvantage lies in the fairly simple treatment of the instrumental resolution function. Only the two parameters *Q*_damp_ and *Q*_broad_ are used to model the effect of a *Q*-dependent resolution function. Even though most PDF beamlines operate at settings that result in a rather broad resolution function, these two parameters are not really sufficient to describe these effects, especially in cases of complex resolution functions like the ones found in neutron time-of-flight diffractometers (Olds *et al.*, 2018 ▸). To deal with complex resolution functions, Tucker *et al.* (2001 ▸) modified the inverse transformation method originally developed by Pusztai & McGreevy (1997 ▸). In this approach, *G*(*r*) is corrected for resolution effects by adapting resolution parameters in direct space. The resolution parameters are constrained by requiring a good match between the experimental diffraction pattern and the Fourier transform of the PDF back into diffraction space. Still, however, this approach calculates the PDF as a summation in direct space.

## 4. Improved PDF algorithm   

As the PDF obtained from the experimental scattering data is the sine Fourier transform of the reduced normalized scattering function, an improvement on all three points raised in the previous section is surprisingly simple. The calculation of the powder diffraction pattern is straightforward, as demonstrated in any Rietveld program. As the calculated intensity in a powder diffraction pattern consists of the purely elastic contribution and is free from any artefacts that are encountered in the experiment, it is also straightforward to convert the calculated intensity of a powder diffraction pattern into the normalized total scattering function *S*(*Q*) (Egami & Billinge, 2012 ▸). *S*(*Q*) is defined as
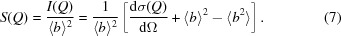
Here *I*(*Q*) is the coherent total elastic intensity and needs to include a term for the thermal diffuse scattering. *S*(*Q*) thus takes the form
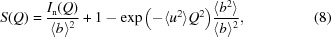
where *I*_n_(*Q*) is the purely elastic powder pattern intensity, normalized by the number of atoms in the model, and 〈*u* ^2^〉 is the average squared atomic displacement. For a periodic structure model, *I*_n_(*Q*) is readily calculated as the sum over all Bragg reflections, including the standard terms on multiplicity and the Debye–Waller terms. The model PDF is readily calculated from equation (8) by the sine Fourier transform of equation (1), which can be implemented as a fast Fourier transform algorithm. As the calculation according to equation (8) is carried out in reciprocal space, the neutron scattering lengths *b* included in this equation are equally well replaced by the *Q*-dependent atomic form factor, which is equally included in the calculation of *I*_n_(*Q*). Equation (8) is thus exact for neutron, X-ray and electron diffraction, at least within the kinematic approximation. Thus, the PDF calculated from a perfectly periodic model structure via the sine Fourier transform of equation (8) is equally exact for all three cases. This is the main advantage of this new algorithm, as implemented in our program *DISCUS* as of Version 6.0 and later.

The calculation of the purely elastic powder pattern intensity *I*_n_(*Q*) allows for several further advantages compared with the traditional PDF algorithm:

(i) Exact result. As detailed in the initial paragraph of this section, the calculation of the powder diffraction intensity in reciprocal space is, within the limits of the kinematic diffraction theory, exact for neutron, X-ray and electron diffraction. For X-ray diffraction this is the main advantage of our new algorithm. Even for electron diffraction, where the use of the kinematic diffraction theory is a severe approximation, the new algorithm will be more reliable.

(ii) Instrumental error check. As our new algorithm initially calculates the diffraction pattern, any errors on the initial *I*(*Q*) data can be checked more reliably. This includes a zero-point offset and wavelength and distance calibration, as detailed by the first example. Identical information would obviously be obtained from a simultaneous Rietveld refinement of the powder data along with the PDF data, which is rarely carried out.

(iii) Computational speed. The calculation of the Bragg intensities is a well established algorithm that allows a fast calculation, even if a high value of *Q*_max_ has been used in the experiment. The computational speed of the PDF calculation becomes independent of the required distance range *r*_max_ in direct space. Only for a calculation over a very short distance range will the traditional direct-space calculation be computationally advantageous with respect to this new reciprocal-space calculation.

(iv) Preferred orientation. Several well tested model functions exist to describe the effects of preferred orientation on the Bragg intensities. These can be used to calculate *I*_n_(*Q*) and will thus describe the effect of preferred orientation on the PDF.

(v) Magnetic scattering. The calculation of the powder diffraction pattern for magnetic structures is also well documented and is implemented in many Rietveld programs. While magnetic scattering, and especially handling of the magnetic PDF, is predominantly the domain of neutron scattering, our new algorithm allows for an easy implementation of both neutron and X-ray magnetic PDF calculations.

(vi) Instrumental resolution. The convolution of the Bragg intensities with an instrumental resolution function likewise is a well established technique, even for instruments with a rather complex *Q* dependence of the instrumental resolution function.

(vii) Sample contribution. The effects of finite size and strain on the widths of the Bragg reflections can also be incorporated into the profile function.

The computational advantages of the PDF calculation via reciprocal space become significantly less if a structure model is to include disorder and thus requires the calculation of both Bragg intensities and diffuse scattering. Such a model requires the simulation of a large supercell in order to represent the disordered structure, while maintaining periodic boundary conditions. If, for example, a supercell of size 20 × 20 × 20 is used, the number of data points that need to be calculated in reciprocal space increases by a factor of 20^3^ = 8000. The computational effort becomes manageable, however, for models that will produce diffuse scattering in limited sections of reciprocal space. As examples, consider materials with stacking faults or materials with 1D disorder, as in host–guest structures with 1D channels. In the case of stacking faults the diffuse scattering is limited to 1D rods, while for 1D disorder in direct space the diffuse scattering is limited to planes in reciprocal space. In these cases the computational increase scales linearly or quadratically, respectively, with supercell size.

As a means of overcoming the huge computational effort in the case of diffuse scattering that is continuously distributed in reciprocal space, we have developed a technique to calculate the PDF via the Debye scattering equation (Debye, 1915 ▸). The details are presented in the *Examples* [Sec sec5] section below.

In the traditional PDF algorithm the width of an interatomic distance distribution is calculated directly according to equation (4). This calculation allows a description of distance-dependent widths, especially for the very first interatomic distance distributions. These are often narrower than distributions at longer distances, since immediate- or second-neighbour atoms tend to vibrate like an acoustic phonon. Thus at any given point in time the distances between the atoms in these pairs tend to be the same, while distances at longer separations will vary as the atoms vibrate independently.

The difference between PDFs calculated with and without distance-dependent parameters consists of small peaks whose intensity quickly decays with increasing distance. The shape of these peaks reflects the difference between the broad peak for the independent vibration model and the narrower peak for the correlated motion model. The position of these difference peaks depends on the actual crystal structure. Since a Fourier transform is additive, the effect of the correlated motion on the diffraction pattern in reciprocal space can be calculated by a sine Fourier transform of the two PDFs back into the reduced total scattering function *F*(*Q*) = *Q*[*S*(*Q*) − 1]. After the Fourier transformation, the two *F*(*Q*) patterns need to be subtracted. Equivalently, the difference PDF can be subjected to the sine Fourier transform. The difference PDF, however, consists of model-dependent peak positions. As a consequence, the difference *F*(*Q*) consists of many model-dependent Fourier components, which cannot be modelled straightforwardly with a single parameter, or even with a few parameters.

Our new PDF algorithm handles the correlated motion effect with the following steps. The normalized intensity is divided by 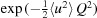. The intensity is next converted into *S*(*Q*) and the sine Fourier transform [equation (1)] is applied to calculate a temporary PDF. The division effectively removes the thermal motion from the model, resulting in a sharpened PDF. The temporary PDF is finally convolved by a Gaussian distribution whose width depends on the interatomic distance according to 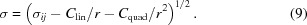
Compared with the traditional width-dependent convolution [see equation (4)], the term 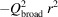 is omitted, as instrumental broadening effects are handled by the convolution of the powder pattern by the appropriate instrumental profile function. The width of the peaks in the temporary PDF is predominantly determined by the value of *Q*_max_. The second example, a silicon data set, will illustrate this part of the algorithm.

## 5. Examples   

In this section we illustrate the new algorithm with five examples. The first two are perfectly periodic materials, CeO_2_ and Si, both collected on beamline 11-IDB at the Advanced Photon Source, Argonne, USA. The third example demonstrates the capabilities for handling preferred orientation. The fourth example is a purely theoretical one to illustrate how this algorithm can be applied to extended supercells. The final example, again a theoretical one, illustrates the capabilities of the algorithm for extended disordered materials.

### 5.1. Crystalline CeO_2_   

**Figure 4:**
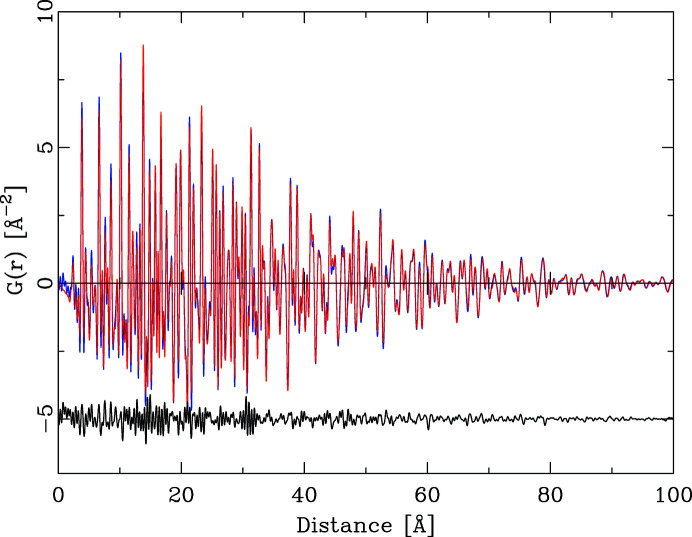
The PDF of CeO_2_ refined with the traditional algorithm. Original data are in blue (background) and calculated data in red. The difference curve PDF_obs_ − PDF_calc_ (black) is offset by −5 Å^−2^ for clarity.

**Figure 5:**
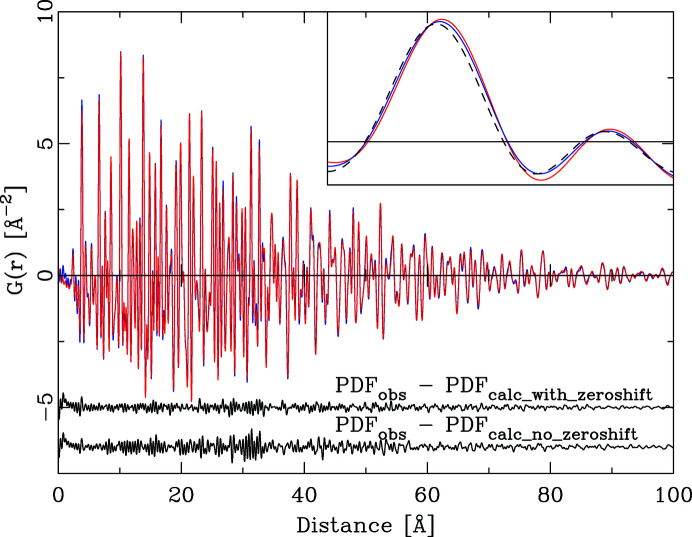
The PDF of CeO_2_ refined with the improved algorithm. Original data are in blue (background) and calculated data in red. The upper difference curve shows PDF_obs_ − PDF_calc_ (black) for the model including a *Q*_zero_ point correction and is offset by −5 Å^−2^ for clarity. The second, lower, difference curve shows PDF_obs_ − PDF_calc_ (black) for the model without a *Q*_zero_ point correction. The inset shows the distance range from 70 to 80 Å; the dashed line corresponds to the PDF calculated without a *Q*_zero_ point correction and is systematically shifted to shorter distances.

**Figure 6:**
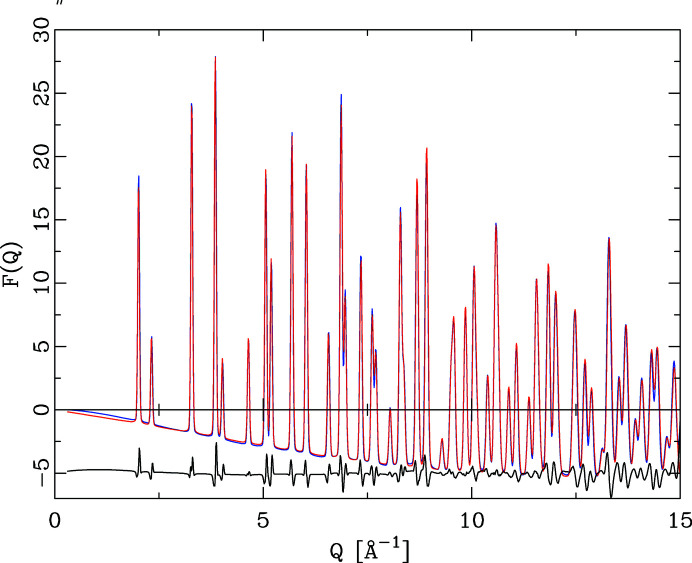
The PDF of CeO_2_ refined with the traditional algorithm, comparing the reduced scattering functions *F*(*Q*). The experimental data (blue) are shifted by Δ*Q* = 0.0024 Å^−1^ compared with the PDF calculated (red) without a *Q* offset. The peak shapes of the difference curve indicate a zero-point error in the experimental data.

The first example uses a data set from CeO_2_ collected on beamline 11-IDB at the Advanced Photon Source, Argonne, USA. Data were collected at room temperature in a capillary geometry in transmission with a wavelength of 0.16 Å and a PerkinElmer area detector. The data were integrated into a 1D powder pattern and transformed into the PDF with *PDFgetx3* (Juhás *et al.*, 2013 ▸). The refinement was carried out with *DISCUS*, using a classical least-squares refinement of the calculated PDF versus the observed PDF. Seven parameters were refined, the ceria lattice parameter, isotropic *B* values for Ce and O, the number density, a scale factor, *Q*_damp_, and *Q*_broad_. The results are given in Table 1 ▸, and Fig. 4 ▸ illustrates the moderate refinement quality (12% weighted *R* value). Identical data were also used to refine a model using the new algorithm. Instead of *Q*_damp_ and *Q*_broad_, five pseudo-Voigt profile function parameters were refined. The mixing parameter of the profile function was defined as η = η + η_L_ *Q*. The common FWHM for the Lorentzian and Gaussian components was defined as FWHM = (*uQ* ^2^ + *vQ* + *w*)^1/2^. Refined values are presented in Table 1 ▸. The improved fit quality is illustrated in Fig. 5 ▸ and is evident in the weighted *R* value of 10.1%. Fig. 6 ▸ illustrates the calculated *F*(*Q*).

**Figure 7:**
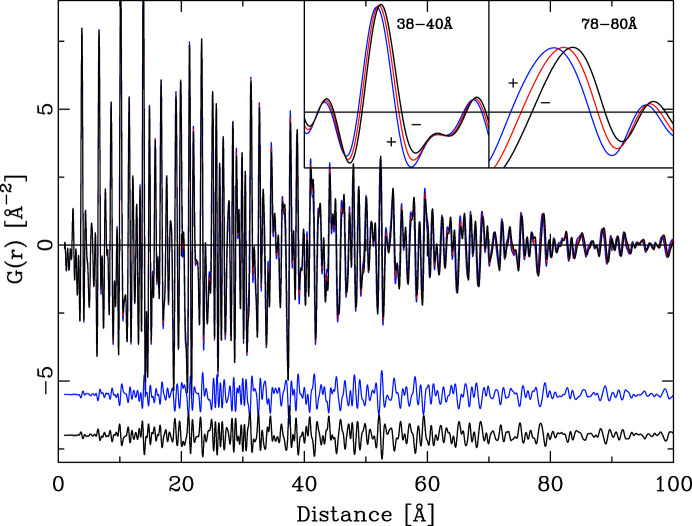
The effect of a *Q*_zero_ shift on the PDF. The (blue) PDF marked with a + was calculated from *F*(*Q*) shifted by Δ = 0.005 Å^−1^ and the (black) PDF marked with a − by Δ = 0.005 Å^−1^. The difference curves are the differences between the ideal PDF (red) and the respective shifted PDF. The effect of a *Q* shift on the PDF is a distance-dependent peak shift.

Note that the data were not refined against *F*(*Q*). As the refinement in the new algorithm proceeds via a calculation of the powder diffraction pattern, it is straightforward to generate the intermittent *S*(*Q*) or *F*(Q*)* values as well. The difference curve in Fig. 6 ▸ indicates as the predominant error a zero-point offset of the original *F*(*Q*) by 0.0024 Å^−1^ compared with the data calculated on the correct *Q* scale. The final refinement included a *Q*_zero_ shift added to the calculated *F*(*Q*) data prior to the conversion into the PDF. This improved the fit quality to 6.7%. With the exception of the profile parameters no parameters shifted significantly. As the new lattice parameter refined to 5.4143 Å instead of the NIST standard value of 5.4116 Å this indicates that the original distance calibration was off by 0.05%. The sine Fourier transform of *F*(*Q*) does not translate the zero-point shift in *F*(*Q*) into a constant distance shift in the PDF. In the absence of any other errors in *F*(*Q*), a distance-dependent peak shift results. The PDFs in Fig. 7 ▸ were simulated for three different *Q*_zero_ shifts. Here, the PDFs for the *F*(*Q*) data set with peaks shifted to larger *Q* are all systematically shifted to a lower distance *r* in the PDF. The distance shift increases with increasing inter­atomic distance *r*, as the peak shift in *F*(*Q*) acts similarly to a pure scale in *Q*, which would result in a pure inverse scale in the PDF. For the actual ceria sample the PDF shifts are less systematic, as further errors are superimposed. Thus, such a zero-point error is not straightforward to detect in the PDF alone.

### 5.2. Crystalline Si   

**Figure 8:**
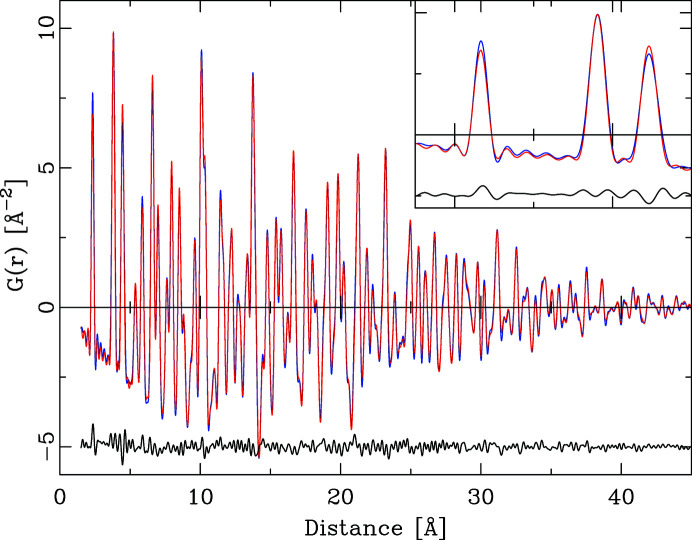
The PDF of Si refined with the traditional algorithm. The experimental PDF is in red and the calculated PDF in blue. The inset shows the distance range 1.5–5.5 Å.

The second example uses a data set from Si, also measured on beamline 11-IDB at the Advanced Photon Source, Argonne, USA. As silicon is a single-atom-type compound the direct PDF calculation is of course exact as well. This example serves to illustrate that the new algorithm can describe the effect of correlated motion just as well as the traditional algorithm. The first peaks in the experimental PDF were analysed with single line fits, resulting in FWHMs of the first three peaks of 0.17, 0.22 and 0.24 Å. Further, the Si—Si distance peaks show the same FWHM of 0.24 Å as well. Figs. 8 ▸ and 9 ▸ illustrate that both refinements result in equally good agreement with the observed data and that the different widths of the first three peaks are described equally well by both algorithms. The three structural parameters [*a*, *B*(Si) and *c*_lin_] are identical within the uncertainties (Table 1 ▸).

### 5.3. Preferred orientation   

The effect of preferred orientation on the intensities in a powder diffraction pattern can be treated by multiplying the intensities of the Bragg reflections with an *hkl*-dependent function, for example the commonly used modified March equation (Dollase, 1986 ▸),
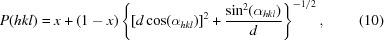
where *x* is the fraction of the sample that is not affected by preferred orientation, *d* is a damping coefficient, and α is the angle between the preferred orientation axis and the reciprocal-space vector represented by *hkl*. The damping parameter *d* describes how sharply the intensity drops as function of α. A more general description is based on the orientation distribution function (Bunge, 1991 ▸; Bergmann *et al.*, 2001 ▸). Currently, no algorithm has been published that allows a direct treatment of preferred orientation of the PDF.

**Figure 10:**
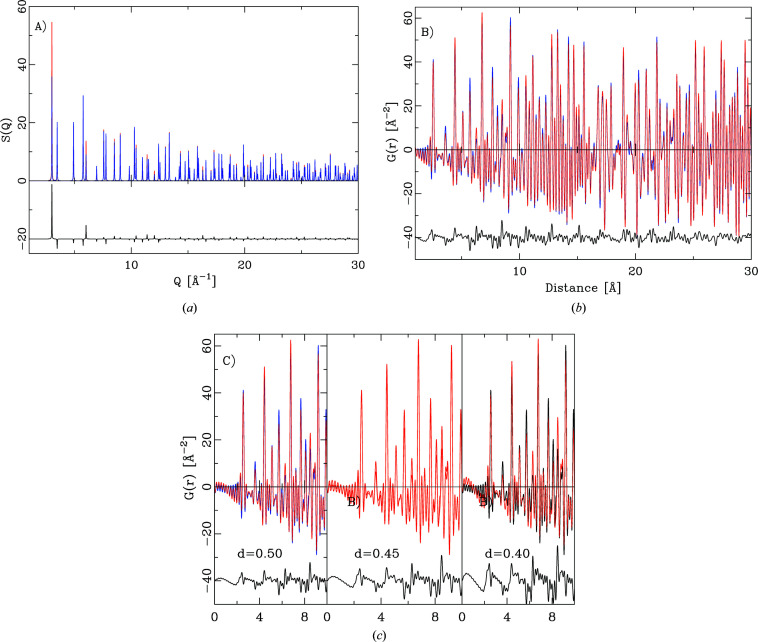
The effect of preferred orientation on the PDF. (*a*) Calculation of *S*(*Q*) for copper without (blue) and with (red) preferred orientation. (*b*) Comparison of the corresponding PDFs. (*c*) Effect of the damping parameter *d*. A decrease in *d* causes an asymmetric background around the PDF peaks.

As an example for our new algorithm, the powder diffraction pattern of copper was simulated without and with preferred orientation [Fig. 10 ▸(*a*)] and the powder pattern transformed into the PDF [Fig. 10 ▸(*b*)]. The preferred orientation axis was chosen as [1, 1, 1], the fraction *x* = 0.6 and the damping coefficient *d* = 0.5. Fig. 10 ▸(*c*) illustrates the effect of the damping parameter *d*. As *d* decreases, the preferred orientation becomes more pronounced. Besides peak height changes, the most prominent change in the PDF is an asymmetric background modulation around the peaks in the PDF.

### 5.4. Application to extended supercells   

The first two examples illustrated the capabilities of the algorithm for perfectly periodic materials. As the main emphasis of the PDF technique lies in the realm of disordered materials we will now illustrate the application of the algorithm to the case of extended supercells.

For this example the initial PDF of a hypothetical perov­skite-type structure, TaSrO_3_ in space group 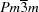, with Ta on 0, 0, 0, Sr on ½, ½, ½ and O on ½, 0, 0, has been calculated using the algorithm described in Section 4. An initial PDF was calculated, based on a single unit cell. The structure was further expanded to a 30 × 30 × 30 supercell, *i.e.* a crystal of 135 000 atoms. This crystal was shaped into a sphere of 100 Å diameter, reducing the number of atoms to 47 785. Using the Debye scattering equation (Debye, 1915 ▸) the powder diffraction pattern of this finite object was calculated. The reduced normalized scattering function for this finite object was converted to the PDF as described in Section 4. As expected, the peak-height PDF of this finite object decreases with increasing distance *r* according to the envelope shape function for a finite object (Howell *et al.*, 2006 ▸; Kodama *et al.*, 2006 ▸),
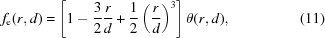
where *d* is the sphere diameter and θ is a step function of value 1 for *r* < *d* and 0 otherwise.

**Figure 11:**
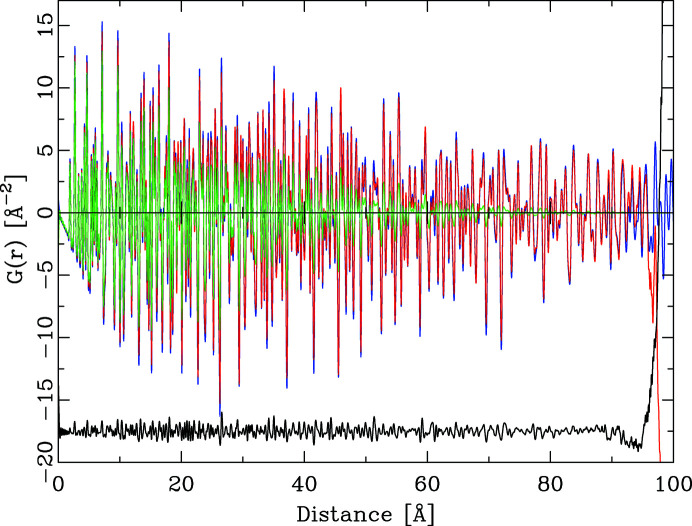
The calculation of a PDF with periodic boundary conditions via the Debye scattering equation. The PDF calculated from a spherical object (green) is divided by the envelope function to yield a PDF (red) that matches the PDF (blue) calculated through the new algorithm up to approximately 80% of the sphere diameter. Beyond 85% the two PDFs differ substantially.

As a final step, the PDF of this finite object was divided by the envelope function of the sphere with diameter 100 Å. This division creates a PDF that corresponds to the PDF calculated from the original single unit cell with periodic boundary conditions, either through an explicit PDF summation in direct space or through the detour via the powder diffraction pattern. Fig. 11 ▸ shows the different calculated PDFs. The difference curve corresponds to the difference between the PDF calculated via the powder diffraction pattern from a single unit cell (blue curve) and the PDF calculated via the Debye scattering equation from the powder pattern of the spherical extended object. The two PDFs are in very good agreement up to approximately 80% of the sphere diameter. Beyond this limit the shape-corrected PDF of the sphere starts to diverge, as the envelope function approaches a value of zero. At shorter distances than the limit of 80%, the differences between the two calculated PDFs are caused by the finite size of the spherical object. Its actual surface is not a perfect sphere but consists of small terraces. Small voids exist between the actual particle surface and the idealized spherical radius that was used to cut the surface. Thus as a function of the distance *r*, the sphere diameter *d* and the actual crystal structure, small deviations remain between the two calculated PDFs.

This concept of creating a PDF with effectively periodic boundary conditions could equally well be applied to any other crystal shape whose envelope function can be calculated analytically. The sphere has the advantage of being a very simple and isotropic object, whose envelope function depends on just a single parameter, the diameter.

Despite the small differences, the new algorithm can very well be used to calculate the PDF of an extended crystal under the assumption of spherical boundary conditions. The algorithm implemented in *DISCUS* allows the user to calculate a PDF that is valid up to distances that correspond to the limit of 80% of the diameter of the largest sphere that can be placed inside the supercell. If the structure within the supercell has been built using any kind of short-range order algorithm, one has to keep in mind that distances longer than 50% of the supercell edge lengths might be subject to aliasing effects.

### 5.5. Disordered perovskite   

In the previous section we showed that our new algorithm can be applied to generate the PDF from a supercell while maintaining the periodic boundary conditions. Here we apply this technique to generate the PDF of a large disordered supercell. The PDF is then refined with a single unit-cell model, once at short distances and then using the PDF data at longer distances.

The supercell for this section was based on the same hypothetical perovskite-type structure TaSrO_3_ as in the last section. The modelling approach is similar to the illustration of the domain concept by Neder & Proffen (2008 ▸). The concept was to create a supercell that consists of domains of a tetragonally distorted perovskite-type structure. Within each domain, the octahedrally coordinated Ta atom at 0, 0, 0 is shifted along one of the three base vectors of the underlying cubic structure. No further distortions of the unit-cell dimensions were applied.

To build the disordered perovskite structure, a primitive unit cell with the perovskite lattice parameters and a single atom at 0, 0, 0 was expanded to a supercell consisting of 43 × 43 × 43 unit cells. All atoms were replaced at equal probabilities by six dummy atom types that each represented a perovskite unit cell with the distortion along the plus or minus direction of one of the three base vectors. These dummy atoms were sorted with positive pair correlations to create reasonably large domains that each consist of a single one of the six atom types. The sorting achieved a final pair correlation coefficient of 0.93. As the structure consists of six different dummy atoms, the probability of finding an identical atom drops below 50% at a distance of 3.5 unit cells, resulting in an average domain size of approximately seven unit cells. Each of the dummy atoms was then replaced by a corresponding complete perovskite unit cell in which the Ta atom at 0, 0, 0 was displaced along one of the three base vectors by a fractional coordinate of 0.1. A sphere of 150 Å was inscribed into the structure and all atoms outside this sphere were removed. The PDF with periodic boundary conditions was calculated from this structure using the algorithm described in the previous section.

Two different single-unit-cell models were refined against this PDF. In the first model only the distance range up to 8.3 Å was used, while for the second model the distance range from 50 to 100 Å was used.

**Figure 12:**
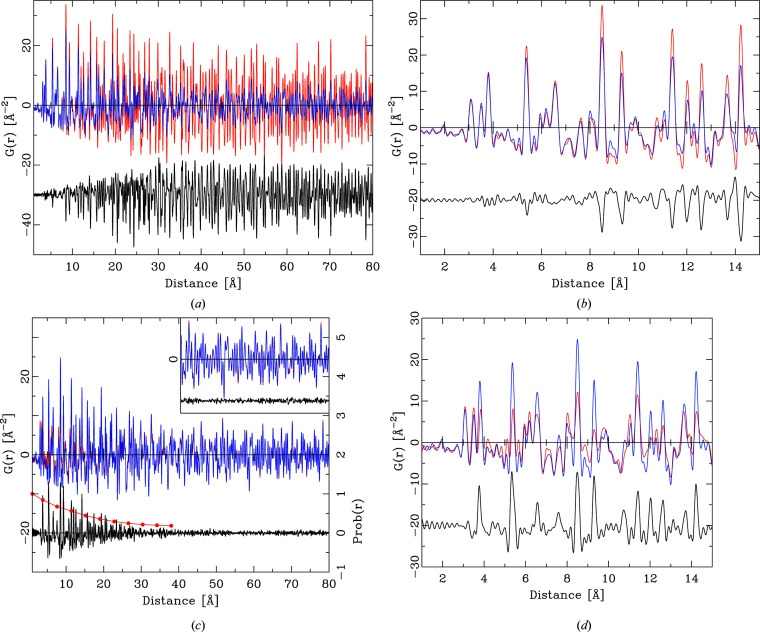
Fits of single-unit-cell models to a PDF simulated from a disordered 43 × 43 × 43 supercell of a perovskite-type structure, TaSrO_3_. (*a*), (*b*) The results of a refinement to the short-distance range up to 8.3 Å. The calculated PDF (red) matches the experimental PDF (blue) for short distances only. (*c*), (*d*) The results of a refinement to the long-distance range 50–100 Å. A good match is observed between the observed PDF (blue) and the calculated PDF (red) in the distance range 50–100 Å. The red dots in panel (*c*) show the probability of remaining within a given domain as function of distance *r*. The trend of the probability scales well with the overall height of the difference PDF (black).

For the short distances one can expect the structure to reflect the structure of a single domain, since on this local level all domains are identical except for their orientation. Accordingly, for the single-unit-cell model the displacement of the Ta atom and independent atomic displacement parameters for all atoms were refined. The scale and profile parameters were fixed to the values used for the initial model building. Table 2 ▸ shows the refined parameters and Figs. 12 ▸(*a*) and 12 ▸(*b*) compare the observed and calculated PDFs. As expected, the PDF calculated using a single-unit-cell model does not describe the PDF at a distance range beyond some 15 Å with acceptable accuracy. At a short-range distance, the fit is perfect up to 5 Å and very good for most peaks up to roughly 10 Å. Those peaks that are dominated by Ta—Ta pairs quickly start to diverge, reflecting the finite size of the domains.

For the long-distance range, the single-unit-cell model must reflect an average of all domains. To this effect, a model structure in space group 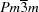 was refined with Ta on an *x*, 0, 0 split position with one-sixth occupancy. As with the short-distance model, independent ADPs were refined while all other parameters were fixed at the input values. Refined values are reported in Table 2 ▸ and the calculated PDFs are shown in Figs. 12 ▸(*c*) and 12 ▸(*d*). Fig. 12 ▸(*c*) shows the excellent fit in the range 50–100 Å and the lack of fit for shorter distances, especially below 20 Å, which is shown in more detail in Fig. 12 ▸(*d*) for distances up to 15 Å.

## 6. Timing results   

The calculation speed of the algorithm depends on the technique that is used to calculate the initial powder diffraction pattern. The timing results reported here were recorded on a PC with an eight-core Intel Xeon E2136 CPU running at 3 GHz.

For the calculation of the PDF via the classical summation in direct space there are three essential steps that affect the CPU time required. In the first step, all partial histograms at all interatomic distances must be established via the double sum over all atom pairs. It scales at least with the atom number squared and to the third power of the maximum distance. In the second step, all partial histograms must be convolved with the distance-dependent profiles that describe the thermal motion and the distance-dependent broadening. This step scales linearly with the number of different pairs and linearly with the maximum distance. In the third step, the partial histograms are summed, and the sum is convolved with the *Q*_max_ termination function and multiplied with the damping function. The overall rate-determining step is the build up of the initial histograms.

For the calculation of the PDF from the content of a single unit cell via the powder diffraction pattern, the steps are the calculation of the powder diffraction pattern, the convolution of the powder pattern by the resolution function, and the normalization and conversion of the powder diffraction pattern into the PDF. The first step scales linearly with the number of atoms per unit cell and to the third power of *Q*_max_, since the number of Bragg reflections scales accordingly. For a fixed *Q*_max_, the number of Bragg reflections scales linearly with the unit-cell volume. As the number of atoms scales linearly with the unit-cell volume (if we assume a constant number density), the calculation scales with the square of the unit-cell volume at a fixed *Q*_max_.

For the calculation of the PDF via the Debye scattering equation, the first step is again the calculation of all partial histograms. Each partial histogram must be transformed and the partial contribution to the powder pattern multiplied with the *Q*-dependent atomic form factors. The next two steps are again the convolution of the powder pattern by the resolution function and the normalization and conversion of the powder diffraction pattern into the PDF. The rate-determining steps are the summing of the histograms and their Fourier transformation.

To estimate the CPU requirements, a single unit cell was used to calculate the PDF up to 100 Å. The number of atoms in the unit cell was modified from 12 to 580 atoms. The (cubic) lattice parameter was scaled to maintain a constant number density. For the standard PDF algorithm, the times required to calculate the PDF and to store the final PDF in the internal software memory ranged from 0.18 to 308 s and are well described by
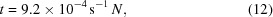
where *N* is the number of atoms per unit cell. For the new algorithm the CPU time is essentially constant at 0.031 s for unit cells with less than 50 atoms. For the larger structures the times increased to 0.21 s and follow
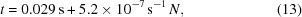
resulting in a computational advantage of three orders of magnitude for the larger structures.

For a comparison of the PDF calculation via the standard summation in direct space and the detour via the Debye scattering equation (DSE), spherical ceria particles of diameter 10–200 Å were simulated. These correspond to objects with 45–369 007 atoms.

For the standard PDF calculation, the times increased from 0.015 to 176 s. For the DSE-type calculations the times were 0.81–184 s. Both trends are well described by
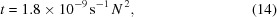
except that the equation for the DSE-type calculation requires an additional offset of approximately 1 s.

Both computations used an identical spherical object. Several other parameters favour the computation time of one method or the other. The constant offset of approximately 1 s in the case of the DSE route corresponds to the overhead due to the Fourier transformation and the convolution of the powder pattern with the profile function. This time is favourably affected by a smaller *Q*_max_ value and increases if the convolution requires a more complex profile function. The time is essentially independent of *r*_max_, as a fast Fourier algorithm is used for the sine Fourier transformation to the PDF. For the standard algorithm, the main rate-determining step besides the computation of the partial histograms is the convolution by the distance-dependent Gaussian function that is used to describe the effect of the thermal motion and the instrumental broadening influence expressed by *Q*_broad_. This is moderately increased by larger displacement parameters and a larger value of *Q*_broad_. In contrast to the calculations with periodic boundary conditions, the calculation is almost independent of *r*_max_.

## 7. Conclusions   

A fast algorithm has been developed that allows a calculation of the PDF that is exact for any type of radiation within the limits of the kinematic scattering theory. As the algorithm calculates the PDF via the sine Fourier transformation of a calculated powder diffraction pattern, any modification to the powder diffraction pattern, such as a convolution with a profile function or treatment of preferred orientation, will properly propagate into the calculated PDF. No restrictions apply to the calculation of the powder diffraction pattern, and examples have been presented for a Rietveld-type calculation of the powder diffraction pattern based on Bragg reflections only, as well as examples based on the calculation via the Debye scattering equation. The first calculation mode allows for very fast PDF calculations and distance-dependent refinements of single-unit-cell models, while the second mode is open to any disordered material.

## Figures and Tables

**Figure 9:**
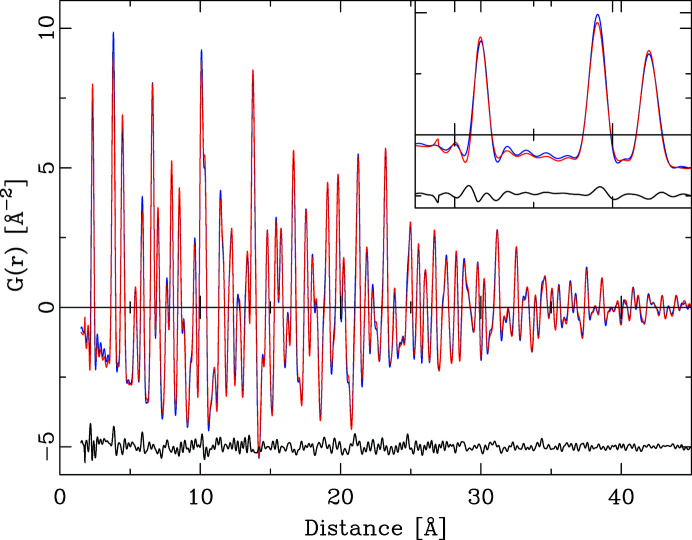
The PDF of Si refined with the improved algorithm. The experimental PDF is in red and the calculated PDF in blue. The inset shows the distance range 1.5–5.5 Å.

**Table 1:** Refined parameters for the different models Columns headed ‘PDF’ refer to calculations with the original algorithm and those headed ‘Powder’ refer to the new algorithm.

	CeO_2_ (PDF)	CeO_2_ (Powder)	Si (PDF)	Si (Powder)
*wR* (%)	12	6.7	6.7	6.8
*a* (Å)	5.4123 (2)	5.4143 (4)	5.3839 (5)	5.3841 (4)
*B*(Ce/Si) (Å^2^)	0.2268 (2)	0.220 (3)	0.529 (2)	0.543 (2)
*B*(O) (Å^2^)	1.552 (1)	0.384 (9)		
*C*_clin_ (Å^3^)			0.0209 (2)	0.0249 (3)
ρ_0_ (Å^−3^)	0.02429 (5)		0.048646 (5)	
Scale	0.3210 (6)	0.2741 (1)	0.949 (9)	0.945 (4)
*Q*_broad_	2.62 (7) × 10^−3^		0.0 (1) × 10^−4^	
*Q*_damp_ (Å^−1^)	2.075 (4) × 10^−2^		0.05672 (8)	
η		0.097 (2)		0.16 (1)
η_L_ (Å^−1^)		−0.006 (1)		
*u*		5.8 (3) × 10^−5^		2.1 (4) × 10^−5^
*v* (Å^−1^)		−1.49 (2) × 10^−4^		−4.1 (8) × 10^−4^
*w* (Å^−2^)		2.42 (5) × 10^−3^		1.97 (3) × 10^−2^
*Q*_zero_ (Å^−1^)		−2.90 (5) × 10^−3^		

**Table 2:** Structural parameters for the perovskite structure for the ideal structure used in the demonstration of the periodic PDF, and the refined values for the model based on the short- or long-range distances

	Ideal	Short	Long
*x*(Ta)	0	0.0960 (4)	0.10009 (1)
*B*(Ta) (Å^2^)	0.1	0.188 (1)	0.1253 (3)
*B*(Sr) (Å^2^)	0.2	0.133 (1)	0.1974 (5)
*B*(O) (Å^2^)	0.3	0.264 (4)	0.355 (1)
